# How to prevent SAM in mitral valve reconstruction

**DOI:** 10.1186/1749-8090-8-S1-O272

**Published:** 2013-09-11

**Authors:** M Cocora, D Nechifor

**Affiliations:** 1 IBCV Timisoara, Timisoara, Romania

## Background

SAM is a life threatening condition with up to 20% risk of sudden death. The present study analyses the mitral reconstructions performed by the authors during the last 5 years, from the point of view of preoperative risk for SAM, the appearance rate of post operative SAM and the ways of dealing with it.

## Methods

We present a retrospective study on 120 cases with degenerative mitral regurgitation in which surgical mitral reconstruction has been tried in the last 5 years. 29 Barlow diseases, 50 FED, 20 idiopathic calcification of PML. Preoperative echographic and intraoperative valve analysis reveals risks factors for SAM; small LV, bulging septum, redundant leaflets, reduced mitro aortic angle, anterior coaptation line. All patients were operated with classical approach, median sternotomy, CEC, standard techniques for mitral valve reconstruction. Surgical methods for SAM prevention are; rings of corresponding dimensions, avoidance of large quadrangular resections, reconstruction of correct ratio between PML and AML dimensions by annular sliding; for large PML prefers the chordae insertion or butterfly resection.

## Results

The incidence in patients with high risk of SAM in the pathology approached was 15%.The incidence of post operative SAM was 3% related to the whole pathology approached or 16% related to the group of patients with high risk of SAM.33% of patients with postoperative SAM were treated by pharmacological maneuvers, the rest requiring surgical reconstruction, with a death rate 25% despite surgical treatment.

## Conclusion

Intraoperative TEE and valve analysis delimitates a group of patients with high risk of post operative SAM. The attention for the prevention of SAM led to the lowering of its incidence. 30% of post operative SAM was efficiently controlled by pharmacological maneuvers.

